# Kinetic photovoltage along semiconductor-water interfaces

**DOI:** 10.1038/s41467-021-25318-8

**Published:** 2021-08-17

**Authors:** Jidong Li, Yuyang Long, Zhili Hu, Jiyuan Niu, Tiezhu Xu, Maolin Yu, Baowen Li, Xuemei Li, Jianxin Zhou, Yanpeng Liu, Cheng Wang, Laifa Shen, Wanlin Guo, Jun Yin

**Affiliations:** 1 Key Laboratory for Intelligent Nano Materials and Devices of the Ministry of Education, State Key Laboratory of Mechanics and Control of Mechanical Structures, Nanjing University of Aeronautics and Astronautics, Nanjing, People’s Republic of China; 2 Institute for Frontier Science, Nanjing University of Aeronautics and Astronautics, Nanjing, People’s Republic of China; 3 Jiangsu Key Laboratory of Electrochemical Energy Storage Technologies, College of Material Science and Engineering, Nanjing University of Aeronautics and Astronautics, Nanjing, People’s Republic of China; 4 State Key Laboratory of Physical Chemistry of Solid Surfaces, Collaborative Innovation Center of Chemistry for Energy Materials (iChEM), College of Chemistry and Chemical Engineering, Xiamen University, Xiamen, People’s Republic of China

**Keywords:** Devices for energy harvesting, Applied physics

## Abstract

External photo-stimuli on heterojunctions commonly induce an electric potential gradient across the interface therein, such as photovoltaic effect, giving rise to various present-day technical devices. In contrast, in-plane potential gradient along the interface has been rarely observed. Here we show that scanning a light beam can induce a persistent in-plane photoelectric voltage along, instead of across, silicon-water interfaces. It is attributed to the following movement of a charge packet in the vicinity of the silicon surface, whose formation is driven by the light-induced potential change across the capacitive interface and a high permittivity of water with large polarity. Other polar liquids and hydrogel on silicon also allow the generation of the in-plane photovoltage, which is, however, negligible for nonpolar liquids. Based on the finding, a portable silicon-hydrogel array has been constructed for detecting the shadow path of a moving Cubaris. Our study opens a window for silicon-based photoelectronics through introducing semiconductor-water interfaces.

## Abstract (web summary)

Common photovoltaic effect is across the interface of heterojunctions. Here, the authors find that scanning a light beam can induce a persistent in-plane photoelectric voltage along silicon-water interfaces, due to the following movement of a charge packet in the vicinity of the silicon surface.

## Introduction

Interfaces of heterojunctions have been a key point of interest in areas from condensed-matter physics^1,2^, electronics^3^, photonics^4^ to electrochemistry^5–7^, owing to plenty of distinctive phenomena and functional devices arising from them. Especially for solid–solid interfaces of semiconductors widely used in electronic devices, broken symmetry therein gives rise to a built-in electric field, which dominates various interesting electronic transport behaviors across the interfaces, such as rectification^8,9^. If charge carriers are excited, the built-in field would pump them out from the interfaces and give rise to an output voltage across the junction, such as that happens in the photovoltaic effect in solar cells. Moving forward to solid–liquid interfaces, electronic transport across interfaces is forbidden for the case without chemical reactions. Instead, the interaction between ion/molecular with the solid plays important roles, as manifested in distinct behaviors of interfacial water^10–13^, ionotronic devices^14–16^ and hydrovoltaic technology^17–20^. Although light illumination on the solid–liquid interface could also excite charges in solid and modulate the solid–liquid interaction, it is challenging to cause continuous voltage output without the assistance of chemical reactions^21,22^. Here, we show that scanning a light beam at the silicon-water interface can induce continuous photovoltage in silicon along the scanning direction, distinguished from normal photovoltage across the junction in photoelectrochemical cells^23–25^. We refer to this effect as a kinetic photovoltaic effect and demonstrated that it can be extended to interfaces between silicon and other polar liquid, such as ethanol.

## Results

**Fig. 1:**
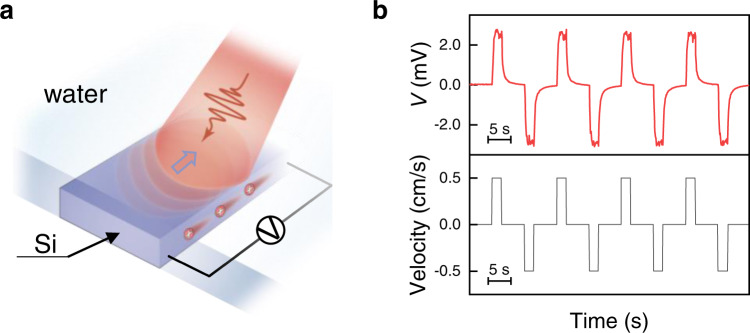
In-plane photovoltage stimulated by scanning a light beam. **a** Schematic illustrating the device structure. In-plane voltage signal across two terminals of a silicon strip in water was recorded while scanning a light beam. **b** Velocity profile of a light beam moving on a p-type Si strip (bottom, black) and the corresponding voltage signal (top, red).

The experimental setup is presented in Fig. 1a with a silicon strip immersed in deionized (DI) water. Voltage signal between two terminals of the silicon strip was recorded while a light beam with a 1-sun intensity and a length of 5 mm, unless otherwise stated, was moved along the silicon-water interface at a controlled velocity, see Supplementary Movie 1. Typical voltage response to the movement of the light beam is depicted in Fig. 1b. When the light beam starts to move at a constant velocity of 0.5 cm/s, the voltage increases gradually to ~2.5 mV in a period around a half second and then fluctuates nearby. It reduces exponentially to the base voltage close to zero once the light beam stops moving. The polarity of the voltage is determined by the moving direction of the light beam, and the terminal at the forward side of the moving-light beam shows a higher electrical potential for p-type silicon.

The voltage polarity is reversed for n-type silicon strip (Supplementary Fig. 1) and its amplitude initially increases with the light intensity and then becomes saturated around 1-sun intensity (Supplementary Fig. 2). Measurements using narrow-spectrum light show that the electrical output decreases significantly for light with a wavelength longer than 900 nm and becomes negligible when it is longer than 1100 nm (Supplementary Fig. 3), consistent with the optical absorption/transmission trend of silicon. Moreover, the silicon strip in air shows no evident response to the movement of the light beam compared to that in water (Supplementary Fig. 4). Controlled experiments also eliminate the possible contribution from pyroelectric (thermal) poling and excited Schottky barrier of the silicon-electrode junction (Supplementary Fig. 5)^26^. These facts imply that the kinetic photovoltaic effect is closely related to the charge excitation of silicon at the silicon-water interface. For the sake of clarity, we focus on the interface between p-type Si and water afterwards.

**Fig. 2:**
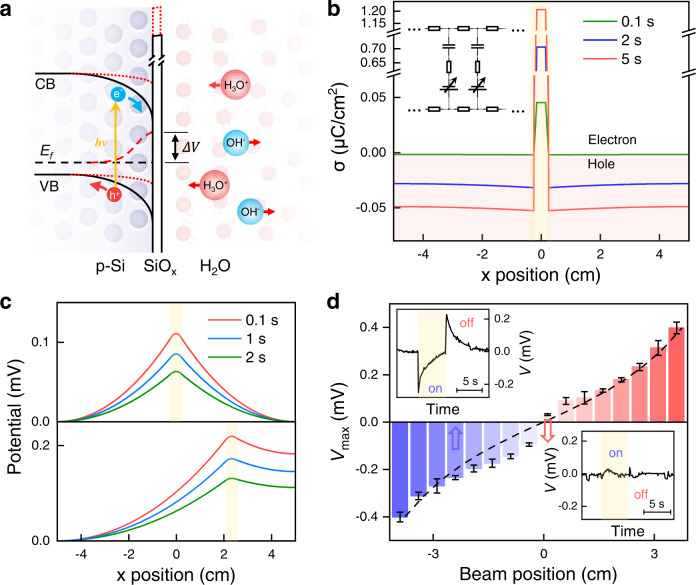
Local charge accumulation induced by light illumination. **a** Schematic of the surface band bending of p-type Si and ion redistribution in the water. VB, CB, *E*_*f*_, and Δ*V* represent valence band, conduction band, fermi level and surface potential change, respectively. The red dashed lines highlight the change of energy band upon light illumination. **b** Time evolution of simulated charge-density distribution along the silicon strip while a light beam illuminates at the center of a silicon strip, as highlighted by the yellow shade. Negative value means the accumulation of holes (as shaded by red color) and positive represents the accumulation of electrons. The left-top inset represents corresponding circuit model (see details in Supplementary Fig. 9). **c** Time evolution of simulated potential distribution while the light beam illuminates at the center (top panel) and off the center (bottom panel), as highlighted by the yellow shades. **d** Dependence of pulsed-light-induced peak voltage on the illuminating position away from the strip center. The vertical error bars indicate standard deviation of the measured peak values. The black dashed line is from numerical simulation. Insets are typical voltage responses to light beam on/off at positions marked by the arrows. The beam-on period is marked by yellow shade and the beam width is 5 mm.

It is well known that a native oxide layer of few nanometers readily forms on and passivates the surface of silicon. Without this oxide layer, the chemical reaction between silicon and water would prevent the generation of kinetic photovoltage (Supplementary Fig. 6). The presence of surface states at the Si/SiO_*x*_ interface causes downward band bending of p-Si^24,27^, as being confirmed by surface potential measurement in air through Kelvin probe force microscopy (Supplementary Fig. 7). The downward band bending gives rise to a depletion region along with a built-in electric field in the vicinity of silicon surface, as illustrated in Fig. 2a.

When becoming in contact with water, the inert Si/SiO_*x*_/water interface showing capacitive behavior without persistent charge transfer across the interface^28,29^, as being confirmed by the electrochemical impedance spectrum (Supplementary Fig. 6). Moreover, photon-induced surface potential change is similar to that in air (Supplementary Fig. 8), indicating that the p-Si surface retains downward band bending. Thus, upon light illumination, photogenerated electrons are pushed forward to the silicon surface due to the built-in electric field, leading to a change in surface potential and charge accumulation upon illumination^30^. At the liquid side, the change of silicon surface potential will drive the motion of dissociated cations of water (H_3_O^+^) toward the surface to screen the accumulated electrons, see Fig. 2a.

Since the Si/SiO_*x*_/water interface is of capacitive behavior, the system can be simplified and described by an equivalent resistor-capacitor (RC) circuit as shown in Supplementary Fig. 9^20^. The Si/SiO_*x*_/water interface is considered as a series of parallel capacitors with the capacitance of *c* in unit area^31^. The change of silicon surface potential upon light illumination is simply reflected by tuning the voltage bias applied to one plate of the capacitors, *V*_0_ for the light illumination region and 0 for the dark region. In this way, charge accumulation in the vicinity of silicon surface upon light illumination can be simulated through the charging of the local capacitors. The circuit was analyzed based on the Kirchhoff laws and then solved numerically through a finite difference method (see Supplementary Note 3 and Supplementary Software 1). The results are shown in Fig. 2b, light illumination on the middle of the silicon strip drives the transfer of electrons from the dark area to the light-irradiated area, giving rise to an electron packet in the middle and hole accumulation in the dark area. The in-plane charge transfer in silicon was also confirmed experimentally (Supplementary Fig. 10).

The redistribution of charges in silicon upon light illumination would give rise to a temporary in-plane current, from which a potential profile can be determined as shown in Fig. 2c, with the highest potential located at the center of the illumination area. If the light beam locates at an asymmetric position, i.e., away from the center of the silicon strip, an asymmetric potential distribution should arise, giving rise to a potential difference between two terminals of the silicon strip. However, static illumination induced in-plane potential gradient would decay rapidly while the charging process becoming saturated.

It is verified by the pulsed-light-induced in-plane voltage as shown in the insets of Fig. 2d. When a light beam located at 2.5 cm away from the strip center is turned on, the in-plane voltage jumps to a peak around 0.2 mV in microseconds and then decays exponentially with a time constant around 1.7 s. An opposite voltage response appears when the light is turned off. A line map along the silicon strip shows that the transient in-plane voltage peak vanishes when the light beam position becomes close to the center, consistent with the simulation results (dashed curve), indicating the validity of this model.

**Fig. 3:**
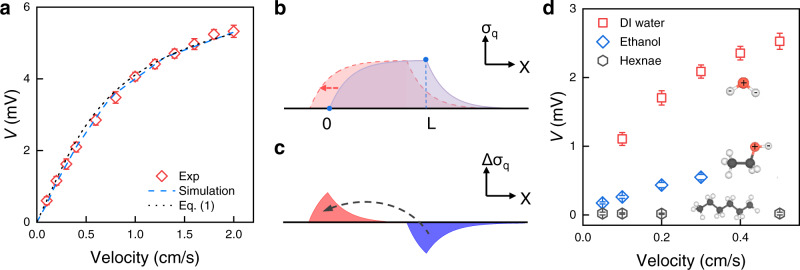
Mechanism of the kinetic photovoltaic effect. **a** Velocity dependence of the moving-light-induced voltage. The black dotted curve is the fit to Eq. (1), and the blue dashed curve is simulation prediction. **b** Blue solid curve and red dashed curve show simplified distribution of accumulated electron density in the vicinity of silicon surface at two moments for a light beam with a width of *L* moving to the left. The initial front and rear edge of the light beam are marked by “0” and “L”, respectively. **c** Difference of the accumulated electron density at two moments, i.e., red shaded area subtracted by blue shaded area in **b**, and the corresponding electron flow direction marked by a dark arrow. **d** Voltage as a function of velocity for p-type silicon in polar DI water, ethanol and nonpolar hexane, respectively. Error bars represent standard deviation. Insets show their molecular structures with labeled charge centers.

To elucidate the origination of the persistent in-plane photovoltage, dynamics corresponding to the movement of the light beam have to be considered. The local voltage bias applied to one plate of the capacitors in the RC circuit was then set as a function of time and position to simulate the scan of the light beam. Simulated waveform of the moving-light-beam induced voltage signal shows the same features as the experimental results (Supplementary Fig. 9b). Moreover, we measured the in-plane voltage response at a series of velocity *u* from 0.1 cm/s to 2.0 cm/s. The total displacement of the light beam, *s*, is fixed at 1 cm during the measurement. The measured maximum voltage output is plotted in Fig. 3a as a function of velocity, which initially increases with the velocity but then becomes saturated. The saturation of the voltage with the velocity is also consistent with the prediction based on the RC circuit, and the *V* - *u* curve predicted by the simulation follows the experimental data well.

To further reveal the mechanism underlying the kinetic photovoltaic effect evidently, a simplified model ignoring hole-distribution background in the dark region is proposed, as the hole density in dark region is much lower than electron density in its accumulated area, see Fig. 2b. At dynamic equilibrium state, distribution of accumulated electrons along the length of the silicon strip approximately follows a piecewise function with exponential dependence on the position *x* (blue shaded area in Fig. 3b) due to the exponentially charging or discharging behavior of the interfacial capacitor. Movement of light beam to the left (negative direction of *x*) requires the following of the charge packet (red shaded area in Fig. 3b). The difference of charge distribution at these two moments, as shown in Fig. 3c, consists of a charge-excess area at the rear and a charge-void area at the front. Then the current induced by the moving-light beam could be considered as the transfer of excess charges at the rear to the front, and the in-plane voltage with consideration of the limited displacement of the light beam can be predicted as (see Methods)

1 $$V={R}_{s}c{V}_{0}L(1-e^{-\frac{{ks}}{u}})u,$$

where *R*_*s*_ is the sheet resistance of silicon strip, which is measured to be around 1 kΩ/sq, *k* is a fitting parameter. Taking the same parameters adapted during the numerical simulation for *c* and *V*_0_, the experimental results can also be well described by the model with a fitted *k* = 1.36 × 10^−6^ s^−1^.

Based on the above discussion, it is clear that local charge accumulation in the vicinity of the silicon surface upon light illumination is essential for the observed kinetic photovoltaic effect. Since charge density is proportional to the interfacial capacitance, i.e., *σ* *=* *V*_0_ × *c*, notable charge accumulation requires the presence of large interfacial capacitance. Thus, polar solvents are preferred due to their higher permittivity. For instance, the relative permittivity of polar water and ethanol at room temperature is around 80 and 24, respectively, significantly larger than that of nonpolar hexane, which is only 1.89. Besides, the dissociation of water and ethanol into charged ions endows the liquid with electrical conductivity, which is essential for the electrostatic induction in liquid and the generation of kinetic photovoltage. Thus, at the same velocity, moving-light-beam induced voltage for silicon in ethanol is more than four times lower than that in DI water due to the lower permittivity of ethanol as seen in Fig. 3d. In contrast, no evident in-plane voltage is observed for nonpolar hexane due to its ultralow permittivity and the lack of dissociated ions.

**Fig. 4:**
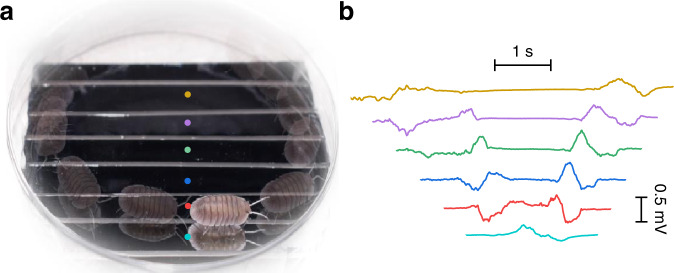
Monitoring shadow path. **a** Photography of a silicon array capped with hydrogel to sense the path of a walking Cubaris. The motion blur records the Cubaris tail, one frame per second. **b** Recorded voltage curves from silicon strips while the Cubaris walks under light illumination of 1-sun intensity. Each colored curve corresponds to the silicon strip marked by a spot of the same color in **a**. Left electrodes of silicon strips are connected to the positive ends of a multi-channel voltmeter.

To explore the potential applications of these effects, we replace liquid water with transparent polyacrylamide hydrogel, which can be seen as water packets embedded in a polymer network with a mesh size of tens of micrometers (Supplementary Fig. 11a). The polymer network makes the hydrogel an elastic solid without disturbing the properties of liquid water^15,32^, thus a solid-state device based on silicon-hydrogel shows a similar response to the moving-light beam. Not only moving-light beams, the in-plane photovoltage can also be induced by shadows of moving objects but with a reversed polarity (Supplementary Fig. 11c), which can be simply regarded as the movement of a charge-poor region in the vicinity of the silicon surface (Supplementary Fig. 11d). Therefore, integrating several silicon strips with a hydrogel film makes it possible to sense the path of a moving object, such as a walking Cubaris demonstrated here. As illustrated in Fig. 4, when the Cubaris strolled on the hydrogel surface from the top left corner to the bottom, a series of voltage signals was induced in the silicon strips due to the asymmetric position of its moving shadow. As the Cubaris arrived one silicon strip, negative voltage across the strip was detected, following a positive signal when it left the silicon strip. When it ran along the silicon strip marked by a red spot in Fig. 4a from left to right, a continual positive signal was acquired before it left the bottom strip. Then it walked from the bottom to the top right corner, giving rise to a series of positive peaks followed by negative ones across each strip. Besides, the kinetic photovoltage shows excellent stability over hundreds of cycles (Supplementary Fig. 12), facilitating its practical applications.

## Discussion

In summary, we revealed a kinetic photovoltaic effect along the silicon-water interface. The presence of charged surface states at the Si/SiO_*x*_/water interface gives rise to a built-in electric field in the vicinity of the silicon surface, which drives the formation of a local charge packet upon light illumination. The following movement of the charge packet due to scanning light beam induces a continuous current and persistent in-plane voltage. In principle, this effect can be extended to inert interfaces between various semiconductors and polar liquids, as well as variation of light illumination besides the moving-light beam and shadows demonstrated here. The variant of these effects could enable a diversity of applications in energy transduction and sensors.

## Methods

### Device fabrication

Single-side-polished Si(100) wafers were purchased from Shanghai Zixi Electronic Co., Ltd and Ningbo Sibranch Microelectronics Technology Co., Ltd with a thickness of ~100 µm and resistivity of 1–20 Ω·cm. The wafers were cut into silicon strips with a width of ~1 cm unless otherwise stated. To make reliable electrical contact of the electrodes, indium particles were pressed onto scratched silicon surface with copper wire embedded.

For the fabrication of silicon strip array integrated with hydrogel, the width of each silicon strip was ~8 mm and the indium electrodes were further sealed by silicone rubber to reduce possible thermal and optical contribution from junctions between silicon and indium. To prepare polyacrylamide hydrogel, 15.6 g acrylamide (Aladdin), 0.0094 g N,N-methylenebisacrylamide (SCR) and 0.0266 g ammonium persulfate (Aladdin) were fully dissolved and mixed in 50 mL DI water^15^. Then 0.039 g crosslinker N,N,N‘,N’-tetramethylethylenediamine (Aladdin) was added for curing. The prepared solution was cast into a glass mold with a ~0.5 mm gap and then solidified overnight. The as-obtained hydrogel film was stored in DI water for use.

### Setup and electrical measurements

A solar simulator (Newport Sol3A) was used to provide AM 1.5G sunlight (100 mW∙cm^−2^). A shading mask was placed closely above the water surface to provide a narrow window that allows a rectangular light beam to pass through and illuminate the silicon surface. A step motor was used to precisely control the velocity of the shading mask. The real-time response of the voltage signal was acquired by DMM6500 with a 10 MΩ internal resistance. A voltage shift may be developed due to the background light and thermal fluctuation. This offset was subtracted from the measured voltage to obtain the kinetic photovoltage originated from light beam movement. The multi-channel voltage of the silicon array was measured by a data acquisition card (NI USB-6281).

### Simplified model

When a light beam scans forward on an infinitely long silicon strip, a charge packet forms and moves forward. Arbitrary position of the silicon strip is presumed to charge (illuminated region) or discharge (dark region) exponentially as *cV*_0_*e*^*-kt*^ where *c* is the interface capacitance per unit area and *V*_0_ is light-induced surface potential change. The equilibrium form of the charge packet under constant velocity *u* is depicted in Fig. 3b and its charge-density distribution *σ* can be written as

$$\sigma =\left\{\begin{array}{cc}0 & {if}\,x\le 0\\ {{cW}{V}_{0}-{cW}{V}_{0}e}^{-\frac{{kx}}{u}} & {if}\,0 \; < \; x\;\le\; L \\ {{cW}{V}_{0}e}^{-\frac{k\left(x-L\right)}{u}}\,& {if}\,x \; > \; L\,\end{array}\right.,$$

where “0” and “L” denote the front and rear edge position of the moving-light beam, and *W* is the width of the silicon strip. The moving of the charge packet leads to current flow in the silicon and its distribution is given by $I={\int }_{0}^{x}\frac{d\sigma {dx}}{{dt}}=u\sigma$. Consequently, potential drop across the silicon strip with an equilibrium charge packet is $\bar{V}={\int }_{0}^{\infty }I{R}_{s}/{Wdx}={R}_{s}c{V}_{0}{Lu}$, where *R*_*s*_ is the sheet resistance of silicon. Before the charge packet gets equilibrium, however, the potential drop across the silicon exponentially increases when the light beam starts to move, i.e.,

$$V=\bar{V}(1-e^{-{kt}})={R}_{s}c{V}_{0}L(1-e^{-\frac{{ks}}{u}})u.$$

## Supplementary information

Supplementary Information

Description of Additional Supplementary Files

Supplementary Movie 1

Supplementary Software 1

## Data Availability

All data needed to evaluate the conclusions in the paper are present in the main text or the Supplementary Information. Additional data related to this paper are available from the corresponding author upon reasonable request.

## Code availability

Custom-written code for the simulation of circuit model is available as Supplementary Software 1.
