# Supplementary material for: Kinetic photovoltage along semiconductor-water interfaces
Source: Nat Commun. 2021 Aug 17;12:4998. doi: 10.1038/s41467-021-25318-8 (PMC8371154; doi:10.1038/s41467-021-25318-8)
Supplement: Supplementary file 1 — Supplementary Information [file 41467_2021_25318_MOESM1_ESM.pdf]

Supplementary Information for

Kinetic photovoltage along semiconductor-water interfaces

Jidong Li[1,2], Yuyang Long[1], Zhili Hu[1], Jiyuan Niu[1], Tiezhu Xu[3], Maolin Yu[1], Baowen Li[1], Xuemei Li[1,3], Jianxin Zhou[1], Yanpeng Liu[1], Cheng Wang[4], Laifa Shen[3], Wanlin Guo*[1,2], Jun Yin*[1]

*Corresponding author. Email: wlguo@nuaa.edu.cn (W.G.); yinjun@nuaa.edu.cn (J.Y.)

[1]Key Laboratory for Intelligent Nano Materials and Devices of the Ministry of Education, State Key Laboratory of Mechanics and Control of Mechanical Structures, Nanjing University of Aeronautics and Astronautics, Nanjing, 210016, P. R. China.

[2]Institute for Frontier Science, Nanjing University of Aeronautics and Astronautics, Nanjing, 210016, P. R. China.

[3]College of Material Science and Engineering, Nanjing University of Aeronautics and Astronautics, Nanjing, 210016, P. R. China.

[4]State Key Laboratory of Physical Chemistry of Solid Surfaces, Collaborative Innovation Center of Chemistry for Energy Materials (iChEM), College of Chemistry and Chemical Engineering, Xiamen University, Xiamen 361005, P. R. China.

Supplementary Notes

Supplementary Note 1: Light sources used for illumination.

Except for simulated solar light, narrow-spectrum light (FWHM ≤ 20 nm) was another light beam source that has been used in our work in Supplementary Fig. 3,5,6,8,10 and 12. It was provided by LEDs for wavelength below 1000 nm and by a xenon lamp with a monochromator for wavelength above 1000 nm. An optical cable was mounted on the step motor to control its movement and guided the narrow-spectrum light onto the silicon surface with a circular spot of ~1 cm diameter.

Supplementary Note 2: Characterization of the Si/SiOx/water interface.

KPFM was performed on the SmartSPM (AIST-NT Inc) integrated with optical spectroscopies. Au coated probe (Multi75GB-G, Budgetsensors) with a spring constant of about 3 N/m was used for surface potential measurements. The topography was scanned and then the tip was lifted 40 nm above the surface to map the surface potential in a tapping mode. A laser spot of 532 nm irradiated on the tip-scanning region and was switched on and off during the mapping.

To study the Si/SiO_x/water interface behavior, electrochemical impedance spectroscopy (EIS) and cyclic voltammetry (CV) measurements were performed on a Biologic VMP-300 electrochemical workstation. Three-electrode configuration was employed, consisting of silicon sample (~1 cm^2) as working electrode, platinum as counter electrode and Ag/AgCl as reference electrode. The oscillating amplitude for EIS measurements was fixed at 20 mV, and the scanning frequency ranges from 7 MHz to 0.1Hz. CV experiments were performed by scanning a range from −1.0 to 1.0 V with a scan rate of 5 mV/s.

Supplementary Note 3: Numerical simulation of light induced signal response.

The simulation is based on the circuit model as shown in in Supplementary Fig. 9. Upon light illumination, the circuit responses with time according to the Kirchhoff laws as

$$i(\mathrm{x},\mathrm{t}) = \frac{1}{\rho_s}\frac{\partial^2 v}{\partial x^2} \tag{1}$$

$$i(\mathrm{x},\mathrm{t}) = -\frac{1}{\rho_w}\frac{\partial^2 w}{\partial x^2} \tag{2}$$

$$\frac{1}{\mathrm{cW}}\mathrm{i} + \frac{1}{\sigma\mathrm{W}}\frac{\partial \mathrm{i}}{\partial \mathrm{t}} = \frac{\partial(\mathrm{v}-\mathrm{w}+\mathrm{u})}{\partial \mathrm{t}} \quad , \tag{3}$$

where W is the width of the silicon strip. The above equations need to be simplified for numerical calculation. Let $f = v - w$ and $\rho = \rho_s + \rho_w$, there is $i = \frac{1}{\rho}\frac{\partial^2 f}{\partial x^2}$. Taking Eq. (1) and (2) to Eq. (3), there is,

$$\frac{1}{\mathrm{cW}\rho}\frac{\partial^2 f}{\partial x^2} + \frac{1}{\sigma\mathrm{W}\rho}\frac{\partial \frac{\partial^2 f}{\partial x^2}}{\partial \mathrm{t}} = \frac{\partial(f+\mathrm{u})}{\partial \mathrm{t}} \quad . \tag{4}$$

The boundary condition is $\frac{\partial f}{\partial x} = 0$, when $x=x_L$ or $x=x_R$. Correspondingly, there is no horizontal

electric current across both boundaries. We use the finite difference method to solve Eq. (4) numerically and adopt an implicit form as $\frac{1}{cw\rho\Delta^2}(f_{i-1}^t - 2f_i^t + f_{i+1}^t) + \frac{1}{\sigma w\rho\Delta^2\tau}(f_{i-1}^{t+1} - 2f_i^{t+1} + f_{i+1}^{t+1} - f_{i-1}^t + 2f_i^t - f_{i+1}^t) = \frac{1}{\tau}(f_i^{t+1} - f_i^t) + \frac{\partial u}{\partial t}$, where Δ and τ are step sizes for x and t. The simulation was implemented with *Matlab*.

Furthermore, combining the Eq. (1) with Eq. (2) yields $w = -\frac{\rho_w}{\rho_s}v + g(t)x + h(t)$. Thus, $f = v - w = v + \frac{\rho_w}{\rho_s}v - g(t)x - h(t)$. We get the potential distribution of silicon as a function of f, i.e. $v = \frac{f+g(t)x+h(t)}{1+\frac{\rho_w}{\rho_s}}$. The boundary condition is $\frac{\partial v}{\partial x} = 0$ *and* $\frac{\partial f}{\partial x} = 0$, if $x = x_L$ or $x = x_R$. So $g(t) = 0$. The in-plane voltage between two terminals of silicon can be calculated via the solved f as $V = v|_{x=\mathrm{xR}} - v|_{x=\mathrm{xL}} = \frac{f|_{x=\mathrm{xR}} - f|_{x=\mathrm{xL}}}{1+\frac{\rho_w}{\rho_s}}$. The simulation was conducted with $\Delta \leq 0.0002$ m and $\tau \leq 0.001$ s. To fit the experimental results in Fig. 3a, ρ_s, W and s are set to 1000 $\Omega\cdot\mathrm{cm}^{-1}$, 1 cm and 1 cm, respectively. In addition, c and V_0 take 30 $\mu F\cdot cm^{-2}$ and 0.2 V according to previous report[1,2]. Parameters are slightly adjusted for the numerical simulation in Fig. 2, where ρ_s, c and V_0 are 500 $\Omega\cdot\mathrm{cm}^{-1}$, 20 $\mu F\cdot cm^{-2}$ and 0.08V, respectively. The circuit model omitted bulk water capacitance and nonlinear effects rising from ion desorption, adsorption and diffusion, which may specially influence its accuracy of high frequency response.

Supplementary Figures

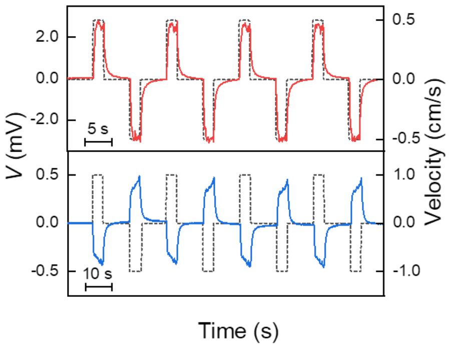

Supplementary Fig. 1| Dependence of voltage polarity on the doping type of silicon. The voltage response of the p-doped (top) and n-doped (bottom) silicon as the light beam scanning periodically forward and backward.

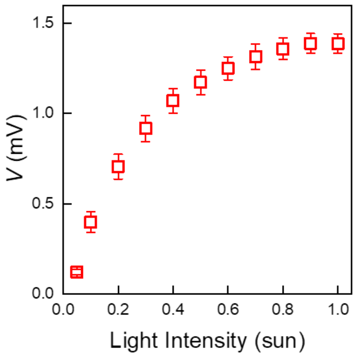

Supplementary Fig. 2| Influence of light intensity on the kinetic photovoltage. The in-plane photovoltage as a function of the light intensity. The velocity of the moving light beam was fixed at 0.5 cm/s. Error bars represent standard deviation.

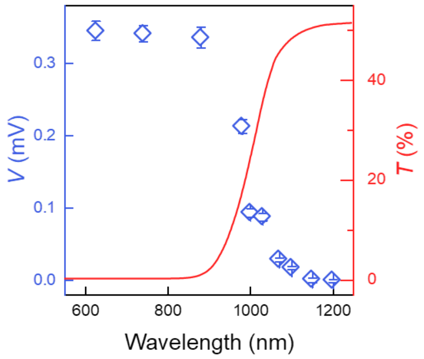

Supplementary Fig. 3| Influence of the light wavelength. The moving-light-beam induced voltage (blue points) and the optical transmission (red curve) of silicon as a function of the wavelength of light. The light intensity for the voltage measurements was fixed around 10 mW. Error bars represent standard deviation of the kinetic photovoltage.

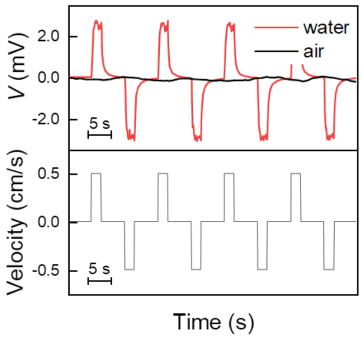

Supplementary Fig. 4| Role of silicon-water interface. The top panel compares the in-plane voltage response of the silicon immersed in DI water and in air while the light beam scans periodically forward and backward. The bottom panel is the corresponding velocity profile of the light beam.

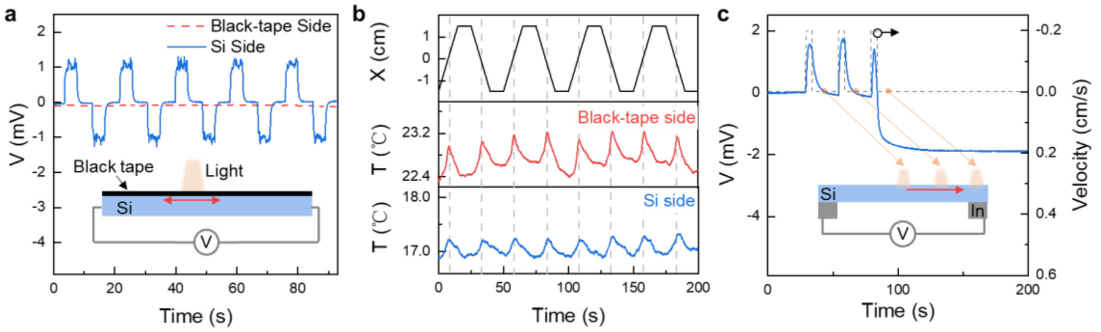

Supplementary Fig. 5 | Contribution from pyroelectric poling and excited Si-In junction. a, The in-plane voltage induced by the light beam scanning on the silicon side (blue solid line) and black-tape-covered side (red dashed line). The black tape is used as light absorber to transfer light beam to heat beam. **b**, The top panel is the position of the light beam center during scanning. A thermal couple was placed at X = 0 cm to record the local temperature while scanning the light beam on the black-tape-covered side (middle panel) and silicon side (bottom panel), respectively. Real time temperature measurement on the sample shows that the local temperature increases several quarters of kelvin while the light beam passes over the position where a thermal couple was placed, and light illumination on the black-tape-covered side give rise to a higher temperature increase due to its higher adsorption efficiency. However, light scanning on the black-tape-covered side does not cause any pronounced change of the in-plane voltage signal, in contrast to the case of light scanning on the silicon side, as revealed in (**a**). These results clearly rule out pyroelectric poling as the possible origination of kinetic photovoltage. **c**, Recorded in-plane voltage (blue solid line) while moving the light beam towards the Si-electrode (n-Si and indium) junction with the velocity profile depicted by black dashed line. In contrast to the decay of voltage once the light beam stops moving, there is a constant photovoltage if the light beam reaching the Si-electrode junction. It indicates that excitation of Schottky barrier of the junction may shift the baseline of measured voltage signal but is not the origination of kinetic photovoltage.

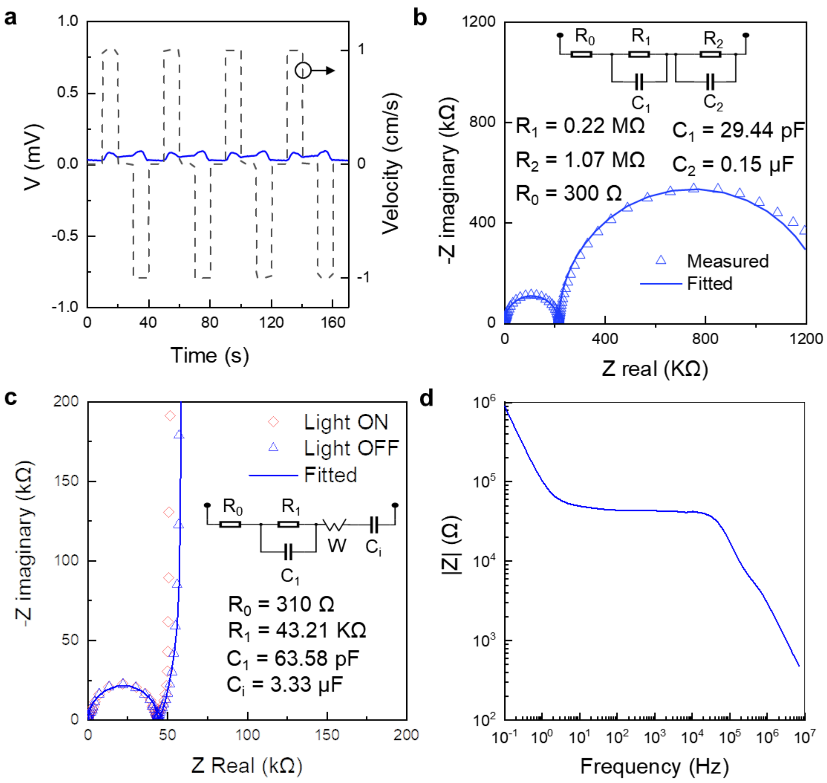

Supplementary Fig. 6 | Impedance spectroscopy of the silicon-water interface and the role of native oxide layer. a, The in-plane photovoltage (blue solid line) recorded across a silicon strip after removing its native oxide layer and the corresponding velocity profile (dashed line) of the scanning light beam. **b**, The impendence Nyquist Plot of a bare Si sample measured with a three-electrode configuration in DI water. **c**, Nyquist plot of a silicon sample without removing the native oxide in DI water, showing almost the same characteristics in the case with and without light illumination (620-625nm, ~15 mW). **d**, Corresponding Bode plot of the light-off data in (**c**). The insets in (**b**) and (**c**) show the equivalent circuit of the impedance spectrum corresponding to the blue fitted curve. R_0 is the internal resistance, including the resistance from both Si sample and its electrical contact. R_1 and C_1 (small capacitance) are attributed to the resistance and geometric capacitance of bulk water[3]. R_2 and C_2 are contributed to the chemical reaction between Si and water. W and C_i represent Warburg impedance and interface double layer capacitor (large capacitance), respectively.

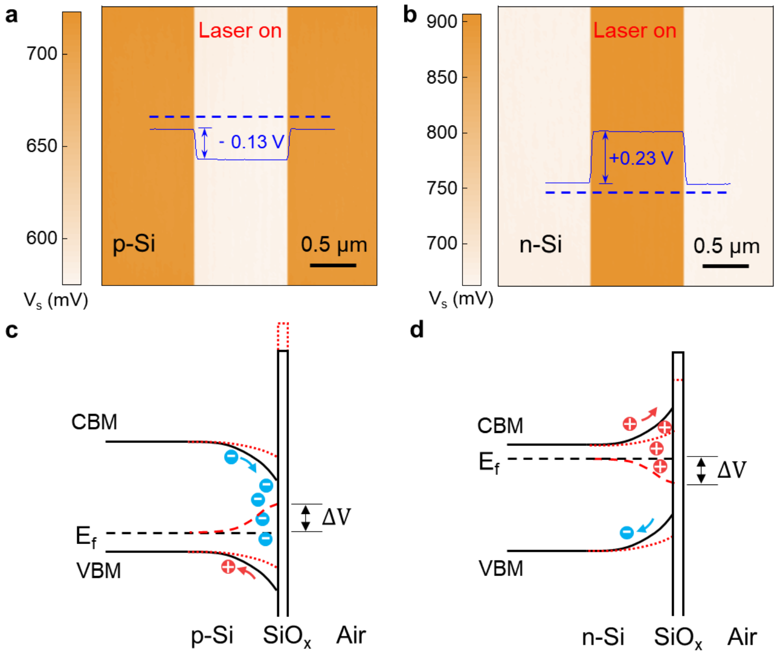

Supplementary Fig. 7 | Dependence of surface potential of Si on light illumination in air. Surface potential of p-Si (**a**) and n-Si (**b**) measured by Kelvin probe force microscopy in air. A laser spot of 532 nm irradiated on the sample underneath the AFM tip while mapping the middle region denoted as 'Laser on'. Inset blue curves are surface potential profiles along the blue dashed lines. **c,d,** Schematics of the band bending of p-Si (**c**) and n-Si (**d**) and their evolution upon light illumination (red lines). VBM, CBM, E_f and ΔV represent valence band maximum, conduction band minimum, fermi level and surface potential change, respectively.

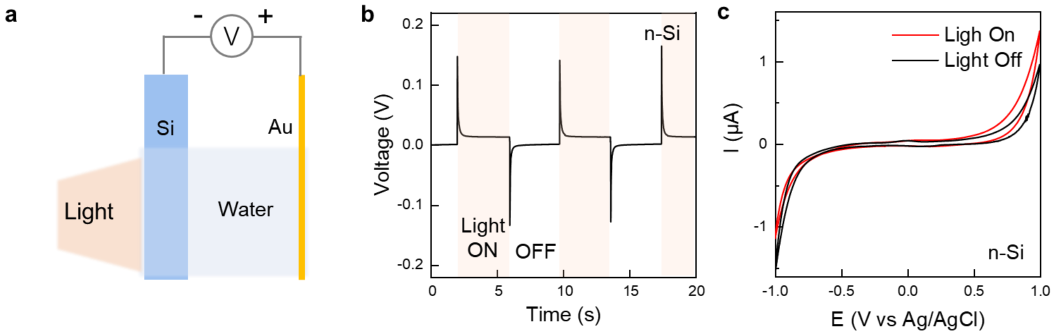

Supplementary Fig. 8 | Surface photovoltage of Si in DI water. a, Schematic of the two-electrode setup for investigating the surface photovoltage of Si in DI water. The working electrode is a Si strip (~100 µm, 1-20 Ω·cm, ~1 cm × 1 cm) and the counter electrode is a gold wire. **b**, Positive voltage pulse appears once the light is turned on for n-Si. **c**, Cyclic voltammetry of n-Si measured in DI water through a three-electrode setup. The I-E curve shifts left upon illumination due to the increase of surface potential.

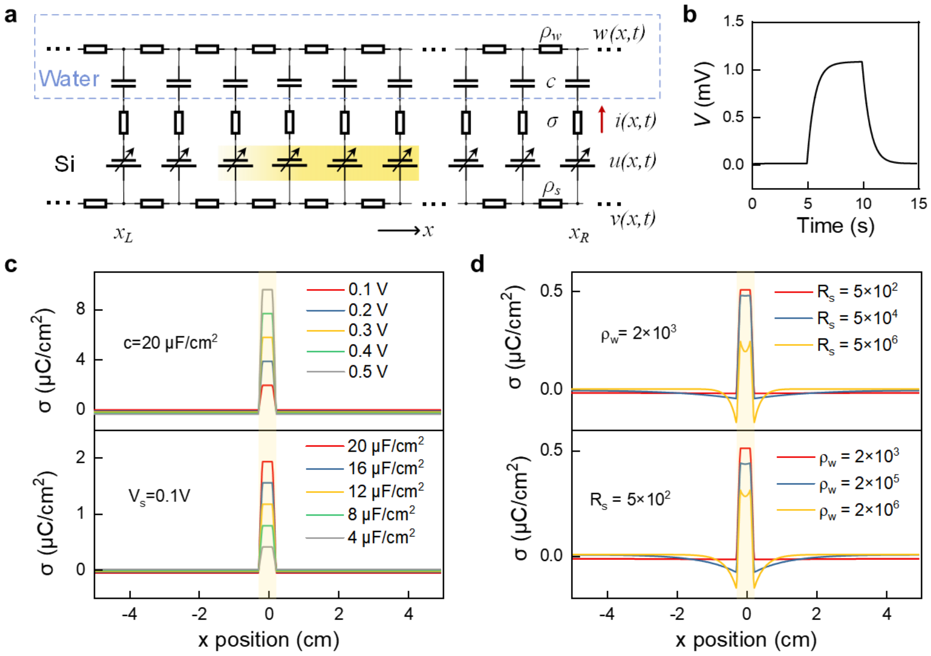

Supplementary Fig. 9| Numerical simulation based on the equivalent circuit model. a, Interfacial capacitance per unit area is denoted as *c*. ρ_s and ρ_w are the resistivity of silicon and water in unit length. *σ and i(x,t)* are the conductance and current across the interface. *v(x,t)* and *w(x,t)* represent the potential distribution in silicon and water. The light illumination and movement can be simulated by varying the bias *u(x,t)*. x_L and x_R represent the negative and positive terminal position of the silicon. **b**, Simulated response of voltage between x_L and x_R induced by a light beam (0.5 cm, length) moving along a silicon strip (10 cm, length). The light beam starts to move at 5 s with a velocity of 0.2 cm/s and stops after 10 s. **c**, Equilibrium charge distribution along silicon while varying the surface photovoltage V_s (Top panel) in the illuminated region and the capacitance *c* in unit area (bottom panel) along the interface. Increasing V_s or *c* promotes the local charge accumulation. **d**, The influence of R_s (top panel) and ρ_w (bottom panel) on the charge accumulation and distribution along the silicon after 1 second of light illumination. R_s (Ω/cm^2) and ρ_w (Ω/cm) are sheet resistance of Si and equivalent resistivity per unit length of water, respectively. Increasing the device resistance, either R_s or ρ_w, will delay the local charge accumulation process and cause non-uniform distribution of charge along the silicon strip. Yellow shades in these figures indicate the light illumination regions.

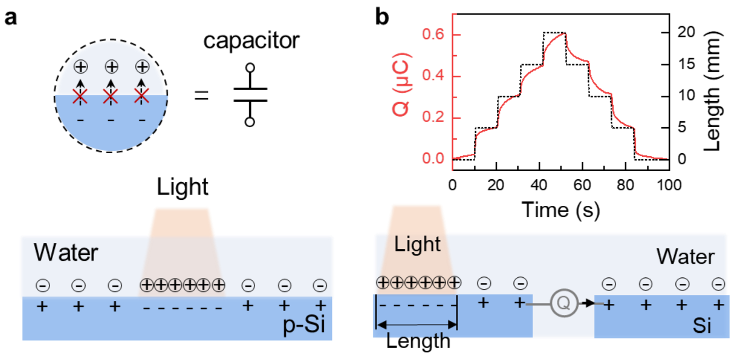

Supplementary Fig. 10 | Charge distribution upon light illumination. **a**, Schematic illustration of the charge distribution at the interface when part of the sample is irradiated by light. **b**, In-plane charge transfer measurement upon illumination, which is illustrated schematically in the lower panel. An electrometer detects the charge flow from the left part of a Si strip to the right part. Both the left and right parts have a length of 5 cm and width of 1 cm. Top panel shows the dependence of accumulated charge in the right part on the Length of light beam. A linear background offset was subtracted.

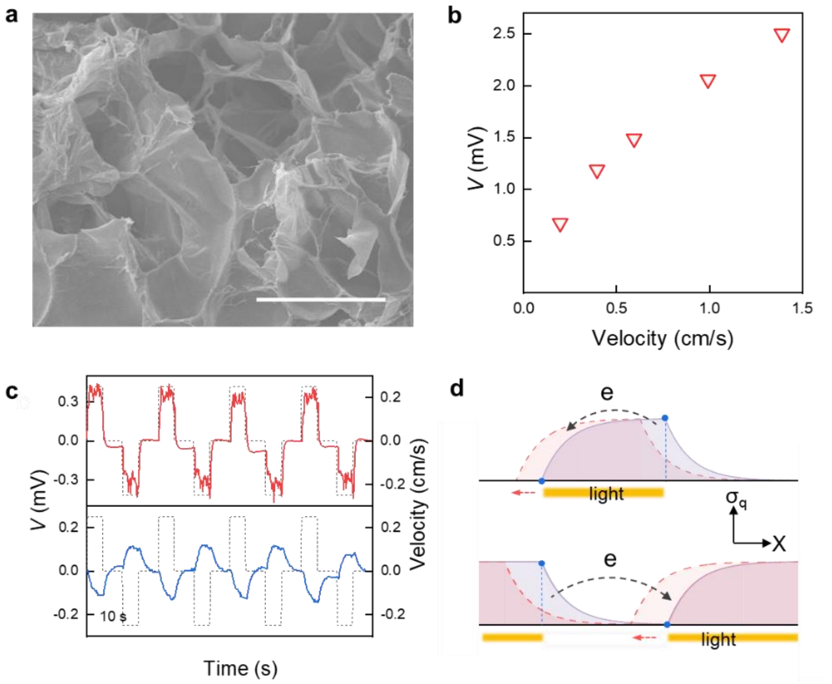

Supplementary Fig. 11| Kinetic photovoltaic effect in a solid device based on a silicon-hydrogel junction. a, SEM image of the hydrogel after freeze-drying treatment showing the polymer network with a mesh size of tens of micrometers for water storage. The scalar bar is 100 µm. **b**, Velocity dependence of the moving-light-induced voltage across a p-type silicon strip covered by a transparent hydrogel film. **c**, Kinetic photovoltage induced by the scanning light beam (top panel) and shadows of moving objects (bottom panel) along the silicon-hydrogel strip. The dashed lines are the velocity profiles. **d**, Blue solid curve and red dashed curve show simplified distribution of accumulated electron density in the vicinity of p-Si surface at two moments for a light beam (top panel) and a shadow (bottom panel) moving to the left, respectively. Blue dots and dashed lines indicate the bright/dark boundaries. Moving of the light beam will drive electron transport from the rear of the light beam to the front, as indicated by the top-panel dashed arrow. Instead, moving of a shadow can be regard as moving of an electron-poor region for p-type Si and will give rise to a reversed transport of electron as indicated by the bottom-panel dashed arrow.

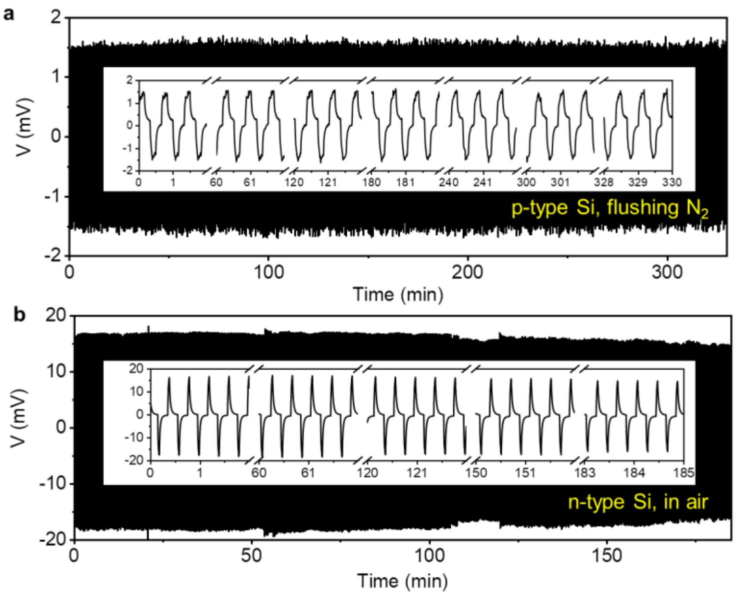

Supplementary Fig. 12| Long-term stability of the kinetic photovoltaic effect. a, Cycling kinetic photovoltage of a p-type Si sample immeresed in DI water with flushing nitrogen gas. **b**, Cycling kinetic photovoltage of a n-type Si in DI water exposed to air. A light source of ~16 mW is provided by a red LED (620-625nm). Scanning velocity is 0.2 cm/s and 0.5 cm/s for (**a**) and (**b**), respectively.

Supplementary References

1. Le, J.-B., Fan, Q.-Y., Li, J.-Q. & Cheng, J. Molecular origin of negative component of Helmholtz capacitance at electrified Pt(111)/water interface. *Science Advances* **6**, eabb1219 (2020).
2. Bastide, S., Gal, D., Cahen, D. & Kronik, L. Surface photovoltage measurements in liquids. *Review of Scientific Instruments* **70**, 4032-4036 (1999).
3. SatyanarayanaRaju, C. & Krishnamurthy, C.V. Charge migration model for the impedance response of DI water. *AIP Advances* **9**, 035141 (2019).
