# Supplementary material for: Kinetic photovoltage along semiconductor-water interfaces
Source: Nat Commun. 2021 Aug 17;12:4998. doi: 10.1038/s41467-021-25318-8 (PMC8371154; doi:10.1038/s41467-021-25318-8)
Supplement: Supplementary file 2 — Description of Additional Supplementary Files [file 41467_2021_25318_MOESM2_ESM.pdf]

Description of Additional Supplementary Files

File Name: Supplementary Movie 1

Description: Experimental setup demonstrating the kinetic photovoltage along semiconductor-water interfaces. A rectangular light beam was formed by projecting a simulated solar source through two rectangular windows, whose scanning motion was driven by a programmed motor. Voltage signal across the silicon strip was recorded with a multimeter and displayed on the front panel.

File Name: Supplementary Software 1

Description: Numerical simulation of illumination-induced signal response along Si/water interface. The numerical simulation was implemented with Matlab and based on the finite difference method as described in Supplementary Note 3. The codes could generate the real-time distribution of potential and charge upon dynamic illumination, which has been used in Fig. 2b-d and Fig. 3a.
